# Chitinases in Tear Fluid of Patients With Amyotrophic Lateral Sclerosis

**DOI:** 10.1111/ene.70757

**Published:** 2026-09-19

**Authors:** Lara Wenz, Lena‐Sophie Scholl, Nya Reinhardt, Ricarda von Heynitz, Vincent Gmeiner, Petra Rau, Philipp Jie Müller, Andreas W. Wolff, Emily Feneberg, Antonia F. Demleitner, Paul Lingor

**Affiliations:** ^1^ Department of Neurology, Klinikum Rechts der Isar, TUM Universitätsklinikum, School of Medicine and Health Technical University of Munich Munich Germany; ^2^ DZNE German Center for Neurodegenerative Diseases Munich Germany; ^3^ Munich Cluster for Systems Neurology (SyNergy) Munich Germany

**Keywords:** amyotrophic lateral sclerosis, biomarker, chitinase, neuroinflammation, tear fluid

## Abstract

**Background:**

Chitinases, including chitotriosidase (CHIT1) and chitinase‐3‐like protein 1 (CHI3L1), are markers of neuroinflammation, a key process in amyotrophic lateral sclerosis (ALS). Tear fluid (TF) can be collected non‐invasively and may represent a promising alternative to CSF or blood to study chitinases.

**Methods:**

TF was collected from 50 ALS patients and 50 control subjects using Schirmer strips. CHIT1 and CHI3L1 levels in TF, serum, and CSF were quantified using ELISA. Serum NfL was measured using SIMOA. The frequency of a 24 bp‐duplication polymorphism in the CHIT1 gene influencing CHIT1 expression was assessed by PCR.

**Results:**

No group differences in the distribution of the CHIT1 polymorphism were detected. Carriers of the polymorphism in both ALS and controls showed lower CHIT1 levels in serum and TF. CHI3L1 levels in TF were higher in ALS patients compared to controls (*p* = 0.007), consistent with changes in CSF but not serum. In ALS, males showed higher TF CHIT1 values compared to females (*p* = 0.009). Combining TF chitinase values with serum NfL values improved discrimination between ALS and controls.

**Conclusions:**

Chitinases are detectable in TF, and CHI3L1 levels recapitulate changes observed in CSF, highlighting its potential for non‐invasive longitudinal assessment. Furthermore, chitinase values in TF, together with serum NfL, may act complementarily by capturing distinct aspects of the disease, neuroinflammation and axonal damage. These results suggest TF chitinases and serum NfL could complementarily contribute to the diagnosis and monitoring of the disease, and call for further evaluation of TF as a biomarker source in ALS.

## Graphical abstract

This study investigated chitinase expression in ALS across tear fluid (TF), CSF, and serum. CHI3L1 was elevated in TF, consistent with changes in CSF, but not serum. Furthermore, combining TF chitinases with serum NfL improved diagnostic discrimination. CHIT1 levels were associated with sex (higher in male TF) and genotype (lower in 24 bp‐duplication carriers). Overall, tear fluid chitinase levels parallel those in CSF rather than serum, reinforcing its potential as a non‐invasive biomarker.

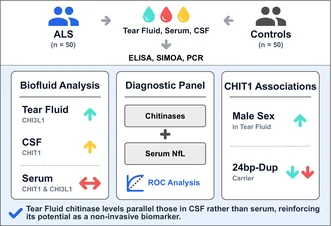

## 1 Introduction

Amyotrophic Lateral Sclerosis (ALS) is a progressive neurodegenerative disorder characterized by the gradual degeneration and death of upper and lower motor neurons, which leads to muscle weakness and atrophy. It typically presents in mid to late adulthood, and the majority of patients succumb to the disease two to five years after symptom onset [1]. The diagnosis of ALS is often delayed and remains challenging due to the considerable heterogeneity of the disease and nonspecific initial symptoms.

Inflammatory processes, such as glial cell activation and infiltration of macrophages and T‐lymphocytes, have been found to be upregulated in the disease course of ALS and are thought to play a key role in its pathogenesis [2]. Clinical trials targeting inflammatory pathways have, however, not been successful in the general population of ALS patients. Recent studies on brain tissue and CSF suggest that inflammatory processes may be the dominant disease mechanism in only a subset of patients [3, 4, 5]. This has further strengthened the view that ALS may represent not a single disease entity but rather a clinical syndrome comprising diverse clinical and molecular subtypes, one of which may be characterized by neuroinflammation [6]. This underlines the urgent need for biomarkers identifying patients with prominent neuroinflammation for clinical interventions and to improve monitoring and outcome measurements of clinical trials.

Neurofilament light chain (NfL) is currently considered the most robust biomarker in ALS, offering reliable prognostic value and outperforming other candidates in distinguishing ALS from healthy controls, mimics, and other neurodegenerative diseases [7]. Nonetheless, its lack of specificity, reflecting general axonal loss rather than specific pathophysiological processes, limits its utility in identifying distinct ALS subtypes. A potential biomarker candidate for the inflammatory subtype is chitinases, which have been shown to be associated with the activity of immune cells like macrophages and, in neurological contexts, microglial and possibly other glial cells [8]. Chitotriosidase (CHIT1) and chitinase‐3‐like protein 1 (CHI3L1) have been found to be increased in the cerebrospinal fluid (CSF) of patients with ALS across multiple studies (reviewed in [9]). In addition, they have been linked to disease progression, disease severity, and patient survival (reviewed in [10]). Results in serum are less conclusive, likely because chitinases are also abundantly expressed in peripheral, non‐brain regions. Furthermore, serum is much more affected by systemic inflammatory processes like asthma and sarcoidosis, thereby reducing its specificity for CNS‐related disease mechanisms [11, 12]. CHIT1 levels in CSF as well as in serum are also influenced by a common 24‐base pair duplication polymorphism in the CHIT1 gene, which leads to lower concentrations in heterozygous and often undetectable levels in homozygous carriers [13, 14].

Collecting CSF is an invasive procedure and can be associated with relevant side effects and thus limits longitudinal assessments. Alternatively, less invasive biofluids like tear fluid (TF) have gained increasing attention as an accessible source of biomarkers for various diseases [15]. Although TF originates as an ultrafiltrate from blood, the lacrimal gland, via parasympathetic innervation, also receives input from regions in the brain stem, which are areas commonly affected in many neurodegenerative disorders. Reduced TF production has been observed across several neurodegenerative diseases, potentially indicating an influence of neurodegeneration in the aforementioned regions on TF production [16]. Furthermore, recent studies have reported changes in expression patterns of established biomarkers in TF of patients with Parkinson's disease, Creutzfeldt‐Jakob disease, and Huntington's disease [17, 18, 19, 20]. Recently, analyses of the TF proteome in ALS patients and healthy controls revealed significant differences in expression using a biomarker signature comprising haptoglobin, serpin C1, and four other proteins [21].

This study aimed to assess CHIT1 and CHI3L1 levels in the TF of patients with ALS and healthy control patients. We correlated the values with those in CSF and serum, as well as clinical and demographic parameters. For assessment of diagnostic performance, we further measured the corresponding serum NfL values and explored the potential additional diagnostic power of chitinases in TF and the other biofluids.

## 2 Materials and Methods

### 2.1 Study Design and Participants

For this monocentric study, patients were recruited between September 2019 and January 2025 at the TUM University Hospital. Samples were collected from 50 patients with ALS who met the Gold Coast criteria [22] at the time of sampling and 50 control subjects. The control group consisted of patients without evidence of neurodegenerative diseases. Patients included in the control group were admitted to the general neurology ward and discharged with the following diagnoses: Peripheral nerve palsy, transient ischemic attack, headache, radiculopathy, vertigo, normal pressure hydrocephalus, muscular dystrophy, carotid stenosis, and spontaneous intracranial hypotension. No other inclusion or exclusion criteria regarding age, sex, disease duration, concomitant diseases, or systemic or topical medication were applied. The disease progression rate for ALS patients was calculated as 48 minus the ALS‐FRS‐R at the time of sampling, divided by the disease duration in months. The characteristics of both groups are shown in Table 1 and Supplementary Table S1 in detail.

**TABLE 1:** Characteristics of the study population.

			Control	ALS	*p*
**Participants**					
	N		50	50	
	Age (y)	Mean ± SD	66.0 ± 11.7	64.8 ± 7.7	0.346^a^
		Median (min–max)	68.5 (43.0–84.0)	64.5 (44.0–83.0)	
	Sex (female)	*N* (%)	20 (40)	25 (50)	0.315^b^
**Clinical data**					
	Wetting length (mm/5 min)	Mean ± SD	25.6 ± 18.0	17.4 ± 17.2	0.016^c^
		Median (min–max)	23.5 (2–70)	12 (2–70)	
	Eye disease (yes)	*N* (%)	9 (18)	11 (22)	0.653^b^
		Missing	1	—	
	Eye medication (yes)	*N* (%)	6 (12)	5 (10)	0.722^b^
		Missing	1	—	
	ALS‐FRS‐R‐Score (points)	Mean ± SD		33.7 ± 7.9	
		Median (min–max)		35.0 (13.0–47.0)	
		Missing		3	
	Disease duration (months)	Mean ± SD		29.6 ± 21.9	
		Median (min–max)		23.0 (4.0–92.0)	
	Progression rate	Mean ± SD		0.7 ± 0.5	
	(−points ALS‐FRS‐R/month)	Median (min–max)		0.5 (0.04–1.86)	
	Vital capacity (%)	Mean ± SD		79.4 ± 24.6	
		Median (min–max)		80.8 (30.1–142.0)	
		Missing		14	
	Onset				
	Spinal	*N* (%)		32 (64)	
	Bulbar	*N* (%)		17 (34)	
	Axial	*N* (%)		1 (2)	
**CHIT1‐polymorphism**					
	Wild‐type	*N* (%)	34 (72)	33 (66)	0.195^b^
	Heterozygous	*N* (%)	13 (28)	13 (26)	
	Homozygous	*N* (%)	0 (0)	4 (8)	
		Missing	3	—	
**Protein—values**					
	Adjusted CHIT1 in TF (arbitrary units)	Mean ± SD	1.53 ± 3.27	1.87 ± 3.51	0.529^a^
		Median (min–max)	0.41 (0.0–16.1)	0.51 (0.0–14.1)	
		< LLOD	14 (28)	17 (34)	
	CHIT1 in serum (ng/ml)	Mean ± SD	48.63 ± 41.08	56.90 ± 62.92	0.831^a^
		Median (min–max)	39.16 (13.47–284.94)	38.25 (0.00–360.00)	
		< LLOD	—	3 (6%)	
	CHIT1 in CSF (ng/ml)	Mean ± SD	6.47 ± 12.79	15.50 ± 10.79	< 0.001^d^
		Median (min–max)	2.10 (0.64–54.22)	11.47 (5.51–37.22)	
		Missing	31 (62%)	41 (82%)	
	Adjusted CHI3L1 in TF [arbitrary units]	Mean ± SD	6.18 ± 4.31	8.22 ± 5.01	**0.013** ^**a**^
		Median (min–max)	4.95 (1.59–22.76)	7.16 (1.58–27.38)	
	CHI3L1 in serum [ng/ml]	Mean ± SD	98.92 ± 200.14	64.17 ± 55.71	0.962^a^
		Median (min–max)	47.07 (16.28–1325.83)	52.05 (14.35–338.85)	
	CHI3L1 in CSF [ng/ml]	Mean ± SD	159.27 ± 67.16	208.48 ± 78.15	0.205^d^
		Median (min–max)	141.35 (47.82–298.13)	198.68 (105.65–338.95)	
		Missing	31 (62%)	41 (82%)	
	NfL in serum [pg/ml]	Mean ± SD	47.29 ± 71.29	79.52 ± 57.11	**< 0.001** ^**a**^
		Median (min–max)	23.10 (5.18–462.00)	70.40 (9.82–214.00)	
		Missing	—	3	

*Note:* TF values were adjusted for total protein concentration.

Abbreviations: ALS, amyotrophic lateral sclerosis; CHIT1, chitotriosidase; CHI3L1, chitinase‐3‐like protein 1; TF, tear fluid; CSF, cerebrospinal fluid; NfL, neurofilament light chain; ALS‐FRS‐R, revised amyotrophic lateral sclerosis functional rating scale; SD, standard deviation.

^a^ Wilcoxon rank‐sum test.

^b^ Pearson's Chi‐squared test.

^c^ Multiple linear regression correcting for age and sex.

^d^ Wilcoxon rank‐sum exact test.

Written informed consent was obtained from all participants. The study complies with the Declaration of Helsinki and was approved by the Ethics Committee of the Technical University of Munich, School of Medicine (approval nos. 9/15S, 2021–473‐S‐KH).

### 2.2 Sample Collection and Processing

TF samples were collected according to a previously published protocol [16] using unanesthetized Schirmer strips (Madhu Instruments Pvt. Ltd., New Delhi, India) (for details, see Supplementary Methods). Wetting length (WL) as well as clinical data (ophthalmological diseases, eye medication, and the use of contact lenses) were noted. Protein isolation was carried out as previously published [21].

CSF and serum were obtained as part of the routine diagnostic work‐up during the same clinical stay as tear fluid sampling (maximum interval ±4 days) and treated according to routine protocols.

### 2.3 PCR Genotyping of the CHIT1 24 Bp Duplication Polymorphism

The patients' DNA was extracted from 3 mL of blood using standard procedures. To detect the CHIT1 24 bp duplication polymorphism, PCR was performed using the following previously published primers: CHIT_ex10 for 5′‐AGC TAT CTG AAG CAG AAG and CHIT_ex10rev for 5′‐GGA GAA GCC GGC AAA GTC, followed by electrophoresis on a 4% agarose gel [23]. The genotype was determined based on the length of the DNA fragments (75 and/or 99 bp).

### 2.4 Biochemical Assays

Chitinase levels in all three biofluids were quantified using commercially available ELISA kits. Kits were tested for efficiency for use with TF. For CHIT1, the CircuLex human chitotriosidase ELISA (MBL, UK) was used according to the manufacturer's instructions. Samples were measured in duplicate with an internal control and diluted 1:2 for TF, 1:50 for serum, and 1:10 for CSF samples. Likewise, for CHI3L1, the human chitinase‐3‐like 1 Quantikine ELISA kit (R&D Systems, UK) was used, and samples were diluted 1:4, 1:50, and 1:200 for TF, serum, and CSF, respectively. For samples with values below the lower limit of quantification (LLOQ) but above the limit of detection (LOD), the value was set at half of the value of the LLOQ, whereas for samples with values below the LOD, the value was set to zero. One control patient showed abnormally high CHI3L1 values in serum (1325.83 ng/mL), almost 30 times higher than the average concentration, and was therefore excluded from all further analyses after testing for outliers using Grubbs' test.

Total protein concentration in TF was determined by BCA to adjust the measured protein levels in TF accordingly.

NfL values in serum were measured according to the manufacturer's instructions using the Simoa NF‐light Advantage Kit, Lot no. 50,3196 (Quanterix, USA).

### 2.5 Statistical and Data Analysis

All statistical analysis was performed using R version 4.4.1 (The R Foundation for Statistical Computing, Vienna, Austria). A *p*‐value below 0.05 (5%) was considered statistically significant. Categorical variables were compared using Pearson's chi‐squared test. For comparisons of continuous variables between two groups, the *p*‐value was determined by the Wilcoxon test, whereas for comparisons with more than two groups, the Kruskal–Wallis test with Dunn's post hoc and Holm correction was used. Prior, all groups were tested for normality by the Shapiro–Wilk test. For comparisons of TF wetting length (WL) between the groups, the cumulative WL of both eyes (in mm/5 min) was calculated, and a multiple linear regression correcting for the confounding variables age and sex was performed. Linear correlations between variables were assessed using Pearson's correlation analysis. Classifier performance was evaluated by receiver operating characteristic (ROC) with the corresponding value of the area under the curve (AUC) and confidence intervals (CI), calculated according to the DeLong method. To account for the discovered sex‐specific differences in CHIT1 expression in TF, all classification models that included TF values were adjusted for sex using multivariable logistic regression. Data are presented as mean ± standard deviation (SD) unless stated otherwise. For visualization, scales were pseudo‐logarithmized.

## 3 Results

### 3.1 Descriptive Analysis of the Study Cohort

TF from a total of 100 patients, of which 50 were ALS patients and 50 controls, were collected and analyzed. Table 1 shows all demographic data as well as biomarker abundance values in detail. Both groups were well matched with regard to age, sex, ocular disease, and medication. Importantly for the CSF analyses, CSF standard parameters such as cell count, glucose, lactate, and albumin ratio were comparable between groups (Table S1). Tear fluid production quantified by wetting length was reduced in the ALS group compared to the control group using a multiple linear regression model correcting for age and sex (ALS mean ± SD = 17.38 ± 17.20 mm/5 min, control mean = 25.60 ± 18.01 mm/5 min, estimate = −8.56, 95% CI [−15.47, −1.64], *p* = 0.016). The wetting length from all patients correlated strongly with the total protein concentration in TF (R_P_ = 0.75, 95% CI [0.64, 0.82], *p* < 0.001, Figure S1).

### 3.2 Analysis of the CHIT1‐24 Bp‐Polymorphism

**FIGURE 1:**
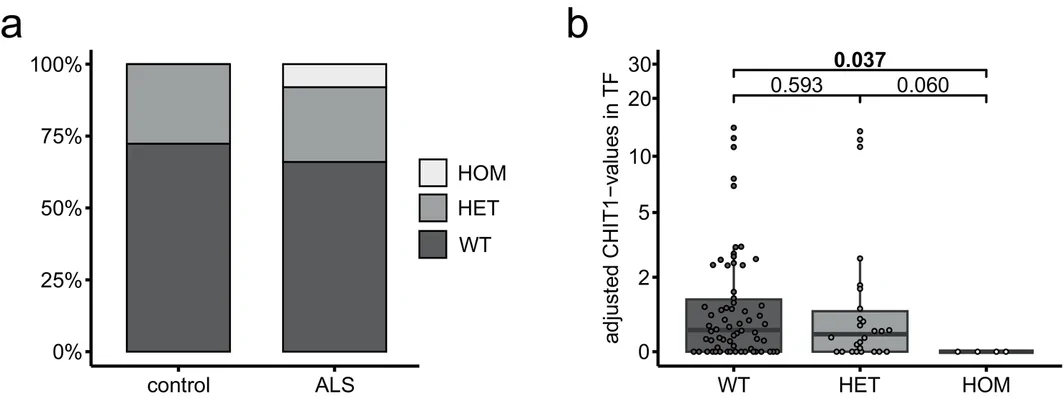
Analysis of the CHIT1 24 bp duplication polymorphism. (a) Distribution of the CHIT1 polymorphism (wild‐type, heterozygous, and homozygous) in the ALS (*n* = 47) and control group (*n* = 50) in per cent. (b) CHIT1 values in TF depending on the CHIT1 polymorphism (wild‐type, *n* = 66; heterozygous, *n* = 36; homozygous, *n* = 4). Subgroups were compared by Kruskal–Wallis test followed by Dunn's post hoc test adjusted for multiple comparisons with Holm correction. Absolute *p*‐values are depicted. Boxplots for all graphs show median and 1st and 3rd quartiles, as well as all individual data points. TF values were adjusted for total protein concentration. Abbreviations: CHIT1, chitotriosidase; CHI3L1, chitinase‐3‐like protein 1; ALS, amyotrophic lateral sclerosis; TF, tear fluid; WT, wild‐type; HET, heterozygous; HOM, homozygous.

We assessed the presence of a 24 bp duplication polymorphism, which has been reported to cause lower expression levels of CHIT1 in heterozygous and homozygous carriers [13, 14]. The control group consisted of 34 wild‐types (72%) and 13 heterozygotes (28%), whereas the ALS group consisted of 33 wild‐types (66%), 13 heterozygotes (26%), and 4 homozygotes (8%). This distribution shows a tendency towards a higher occurrence of the mutation in the ALS group (Figure 1a).

In the serum of carriers of the polymorphism, both heterozygotes and homozygotes showed significantly lower CHIT1 expression levels compared to wild‐types (heterozygotes mean = 47.03 ± 69.69 ng/mL, homozygotes mean = 6.81 ± 13.63 ng/mL, wild‐type mean = 56.64 ± 46.52 ng/mL, p_WTvsHET_ = 0.006, p_WTvsHOM_ = 0.001, Figure S2). The expression levels between heterozygotes and homozygotes also showed a tendency towards lower levels in the latter, close to significance (p_HETvsHOM_ = 0.062).

Similarly, in TF, wildtype carriers showed the highest expression of CHIT1 and differed significantly from the levels of homozygous carriers, although not from heterozygous carriers (wildtype mean = 1.53 ± 2.84, homozygotes mean = 0.00 ± 0.00, heterozygotes mean = 1.88 ± 3.91, p_WTvsHOM_ = 0.037). Like in serum, heterozygous carriers also showed a trend towards higher levels of CHIT1 compared to homozygous carriers, though this difference did not reach significance (Figure 1b).

### 3.3 Expression Levels of CHIT1 and CHI3L1 in All Three Biofluids

The concentrations of the two chitinases, CHIT1 and CHI3L1, were determined in all three biofluids (TF *n* = 100, serum *n* = 100, CSF *n* = 28). TF values of CHIT1 or CHI3L1 did not significantly correlate with the corresponding values in serum or CSF (Supplementary Figure S3a–d). Likewise, no significant correlation was observed between serum and CSF values (Supplementary Figure S3e,f).

**FIGURE 2:**
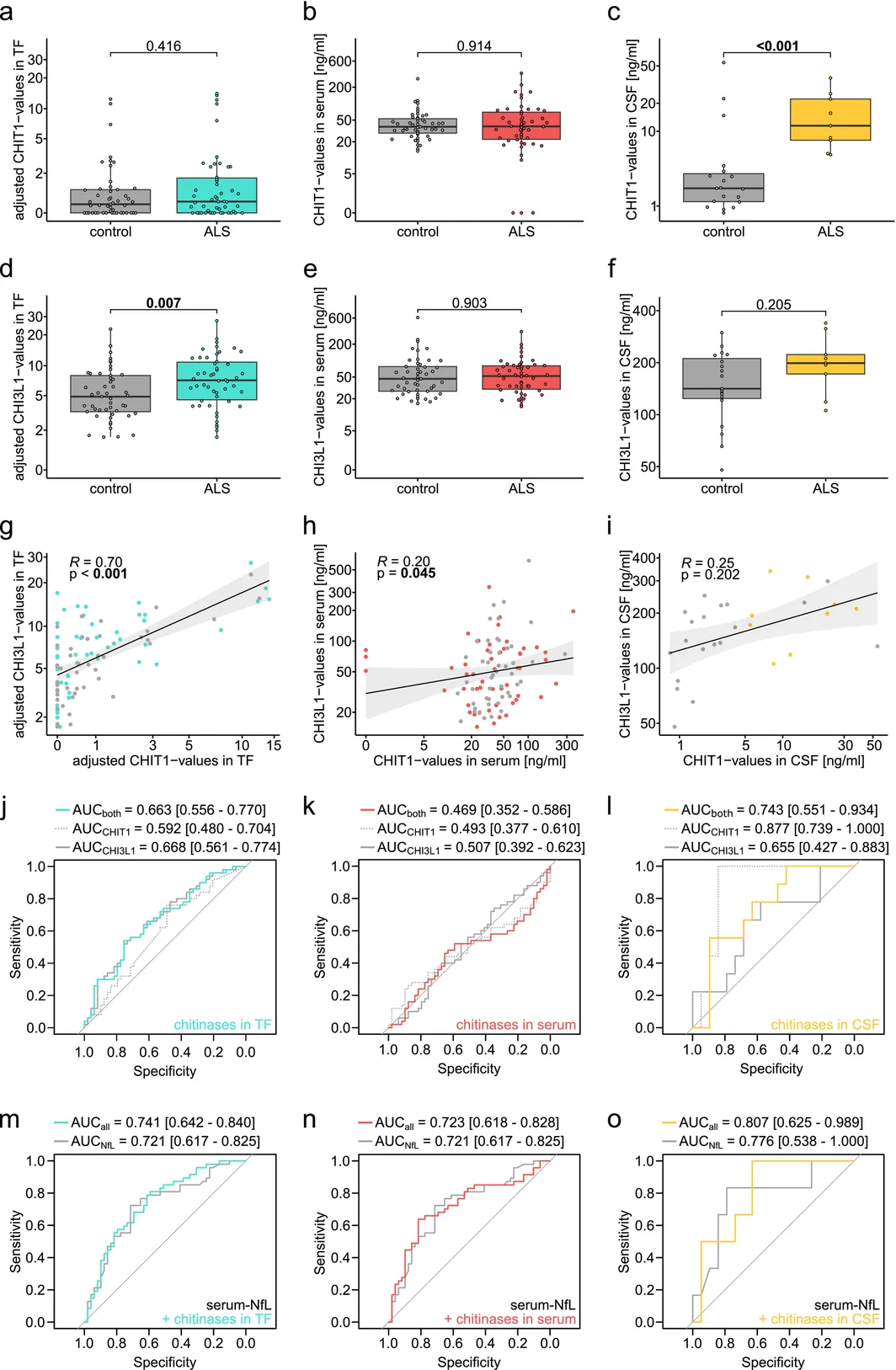
Comparison of chitinase levels in ALS patients and controls in TF, serum, and CSF. (a–c) CHIT1 levels of ALS patients (a = turquoise, b = red, c = yellow) and controls (gray) in TF (a; ALS *n* = 50, control *n* = 49), serum (b; ALS *n* = 50, control *n* = 49), and CSF (c; ALS *n* = 9, control *n* = 19). Boxplots for all graphs show the median and 1st and 3rd quartiles as well as all individual data points. (d–f) CHI3L1 levels of ALS patients (d = turquoise, e = red, f = yellow) and controls (gray) in TF (d), serum (e), and CSF (f). (g–i) Pearson's Correlation of CHIT1 and CHI3L1 values in TF (g), serum (h), and CSF (i). The regression line is shown in black with a 95% confidence interval, as well as all individual data points. Pearson's correlation coefficient was calculated and is depicted at the top with corresponding *p*‐values. (j–l) ROC analysis (ALS vs. controls) of the CHIT1 (dotted gray line) and CHI3L1 values (continuous gray line) separately, as well as both together in TF (j, turquoise line), serum (k, red line), and CSF (l, yellow line). (m–o) ROC analysis (ALS vs. controls) of NfL values in serum (gray line) as well as in combination with both chitinase values in TF (m, turquoise line), serum (n, red line), and CSF (o, yellow line). AUC values with the 95% confidence intervals are depicted. All ROC analyses involving TF values were adjusted for sex using a multivariable logistic regression model. Groups were compared by Wilcoxon test/Mann–Whitney U test. Absolute *p*‐values and AUC values are depicted. TF values were adjusted for total protein concentration before all analyses. Abbreviations: CHIT1, chitotriosidase; CHI3L1, chitinase‐3‐like protein 1; NfL, neurofilament light chain; ALS, amyotrophic lateral sclerosis; TF, tear fluid; CSF, cerebrospinal fluid; ROC, receiver operating characteristic; AUC, area under the curve.

TF concentration of CHIT1 did not differ significantly between the ALS and control groups, although concentrations were numerically higher in the ALS group (ALS mean = 1.87 ± 3.51, control = 1.23 ± 2.53). In CSF, CHIT1 levels of the ALS group were significantly higher than those of the control group (ALS mean = 15.50 ± 10.79 ng/mL, control mean = 6.47 ± 12.79 ng/mL, *p* < 0.001), while in serum no difference was found (ALS mean = 56.90 ± 62.92 ng/mL, control = 48.06 ± 41.30 ng/mL, Figure 2a–c).

CHI3L1 in the TF of ALS patients was significantly higher compared to healthy controls (ALS mean = 8.22 ± 5.01, control = 5.96 ± 4.06, *p* = 0.007). Similarly, in CSF, ALS patients showed numerically, yet not significantly, higher values in CHI3L1 (ALS mean = 208.48 ± 78.15 ng/mL, control = 159.27 ± 67.16 ng/mL). In serum, no difference was detectable between the ALS and control groups (ALS mean = 64.17 ± 55.71 ng/mL, control mean = 73.88 ± 94.28 ng/mL, Figure 2d–f).

The correlation of CHIT1 and CHI3L1 values in TF was strong and significant, whereas it was weak and significant in serum. No significant correlation was found in CSF, although the number of available samples was markedly lower in the CSF group (TF: R_P_ = 0.70, 95% CI [0.59, 0.79], *p* < 0.001; serum: R_P_ = 0.20, 95% CI [0.00, 0.38], *p* = 0.045; Figure 2g–i, Table S1). Neither of the chitinases in TF correlated with any of the clinical parameters, namely the ALS‐FRS‐R score, vital capacity, disease duration, or progression rate (Figure S4).

Subsequent ROC curve analyses were performed, with TF‐based models adjusted for sex to account for the expression differences, as detailed below. Here, CHI3L1 in TF reached an area under the curve (AUC) of 0.668 for the discrimination of ALS from control patients, whilst CHIT1 achieved an AUC of 0.592 (CHI3L1: 95% CI [0.561, 0.774], CHIT1: 95% CI [0.480, 0.704]). The AUC of both chitinases together in TF did not outperform that of only CHI3L1 (AUC = 0.663, 95% CI [0.556, 0.770], Figure 2j). Neither the AUC‐values of the chitinases separately nor together in serum outperformed the values in TF (CHI3L1: AUC = 0.507, 95% CI [0.392, 0.623], CHIT1: AUC = 0.493, 95% CI [0.377, 0.610], both: AUC = 0.469, 95% CI [0.352, 0.586], Figure 2k). CSF outperformed both other biofluids with CHIT1 as well as both chitinases together, whereas for CHI3L1 the AUC‐value was below that of TF (CHIT1: 0.877, 95% CI [0.739, 1], both: AUC = 0.743, 95% CI [0.551, 0.934], CHI3L1: AUC = 0.655, 95% CI [0.427, 0.883], Figure 2l).

### 3.4 Association Between Chitinase Expression Values and Serum‐NfL Values

NfL was measured in the serum of all but three ALS patients. Neither of the chitinases in TF nor serum correlated with the NfL values in serum. In CSF, however, both chitinases showed a strong and significant correlation (CHIT1: R_P_ = 0.64, 95% CI [0.33, 0.83], *p* < 0.001, CHI3L1: R_P_ = 0.57, 95% CI [0.23, 0.79], *p* = 0.003, Supplementary Figure S5).

NfL in serum reached an AUC of 0.721 to distinguish ALS patients from controls (95% CI [0.617, 0.825]). In combination with the sex‐adjusted expression values of both chitinases in TF, the performance was marginally improved to 0.741 (95% CI [0.642, 0.840]), whereas with the chitinase values in serum, it remained largely unchanged (AUC = 0.723, 95% CI [0.618, 0.828], Figure 2m,n). Considering only the patients with available CSF data (*n* = 28), serum NfL performed better (AUC = 0.776, 95% CI [0.538–1.000]). Similarly, the combination of the values of both chitinases in CSF showed an improvement in AUC (AUC = 0.807, 95% CI [0.625–0.989], Figure 2o).

### 3.5 Sex Differences in TF

**FIGURE 3:**
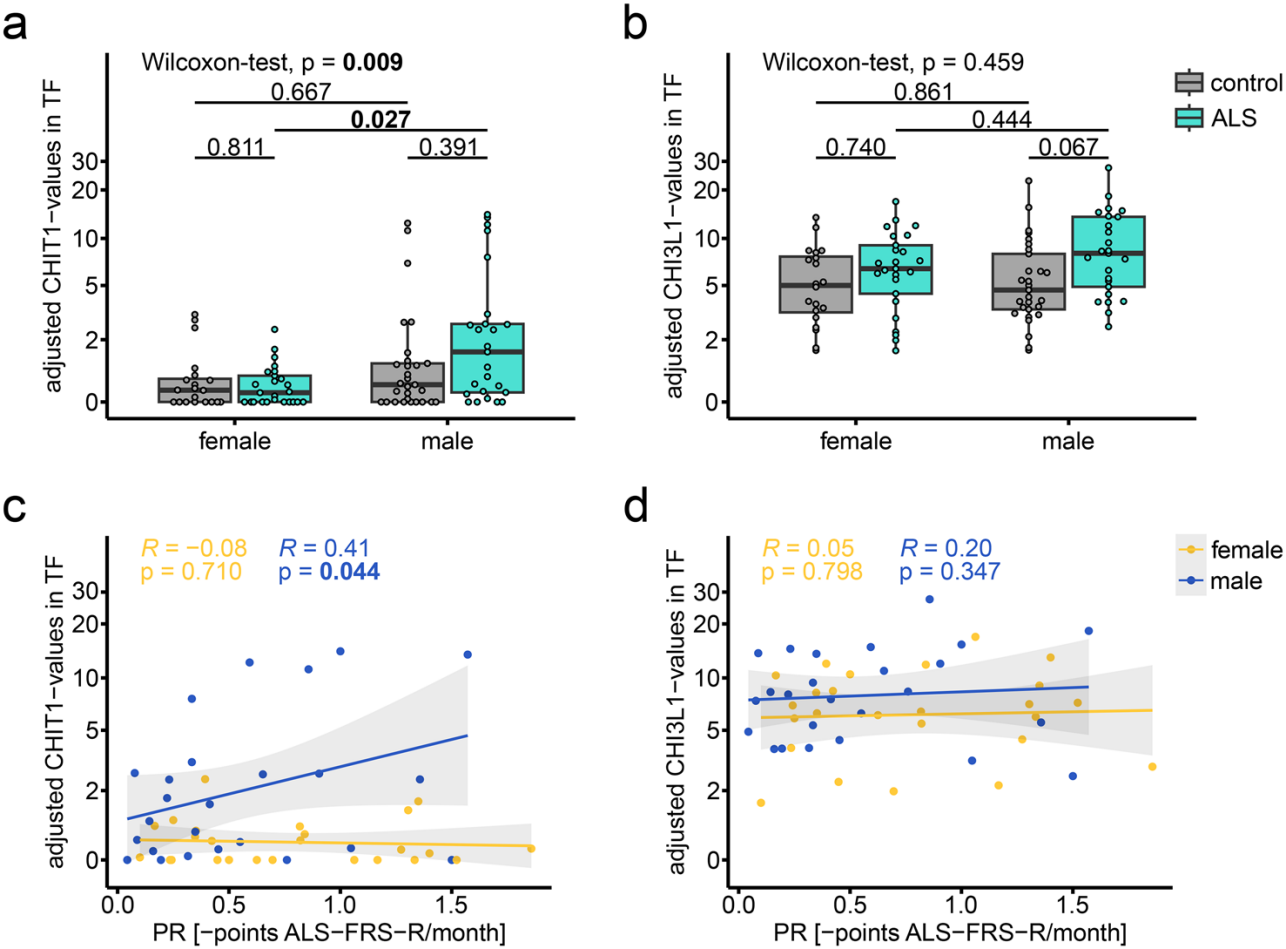
Sex differences in chitinase levels and correlations with progression rate. (a, b) CHIT1 values (a) and CHI3L1 values (b) in TF in males and females, further separated into the ALS (turquoise; female *n* = 25, male *n* = 25) and control (gray; female *n* = 20, male *n* = 29) groups. Subgroups were compared by Kruskal–Wallis test followed by Dunn's post hoc test adjusted for multiple comparisons with the Holm correction. Absolute *p*‐values are depicted (overall sex comparison by Wilcoxon test at the very top, subgroup comparisons by Kruskal–Wallis test with Dunn's post hoc and Holm correction below). Boxplots for all graphs show the median and 1st and 3rd quartiles, as well as all individual data points. (c, d) Pearson's correlation of the progression rate of ALS patients and CHIT1 values (c) and CHI3L1 values (d) in TF stratified by sex. The regression line is shown in blue (males) and yellow (females) with a 95% confidence interval as well as all individual data points. Pearson's correlation coefficient was calculated and is depicted at the top with corresponding *p*‐values. TF values were adjusted for total protein concentration. Abbreviations: CHIT1, chitotriosidase; CHI3L1, chitinase‐3‐like protein 1; ALS, amyotrophic lateral sclerosis; TF, tear fluid; ALS‐FRS‐R, revised amyotrophic lateral sclerosis functional rating scale.

The CHIT1 values in the TF of the male subgroup in the whole cohort as well as in the ALS subgroup were significantly higher compared to the female subgroup (overall male mean = 2.38 ± 3.91, overall female = 0.56 ± 0.80, *p* = 0.009; ALS male = 3.26 ± 4.55, ALS female = 0.48 ± 0.62, *p* = 0.027; control male = 1.63 ± 3.15, control female = 0.66 ± 0.99, Figure 3a). This difference was not significant for CHI3L1 values. Here, the male subgroup showed numerically higher expression levels between ALS and control patients compared to the female subgroup (overall male mean = 7.65 ± 5.38, overall female = 6.45 ± 3.63, ALS male = 9.35 ± 5.83, ALS female = 7.09 ± 3.84, control male = 6.17 ± 4.57, control female = 5.65 ± 3.28, Figure 3b). In the ALS subgroup, CHIT1 values also correlated significantly with the progression rate of ALS in males, whereas the CHIT1 values in females and neither of the CHI3L1 subgroups showed any correlation (R_P_ = 0.41, 95% CI [0.01, 0.69], *p* = 0.044, Figure 3c,d). Moreover, CHIT1 and CHI3L1 separately as well as together performed better in distinguishing between ALS patients and healthy controls in the male subgroup compared to the female (CHIT1: AUC_male_ = 0.632, 95% CI [0.480, 0.783], AUC_female_ = 0.514, 95% CI [0.344, 0.684]; CHI3L1: AUC_male_ = 0.697, 95% CI [0.555, 0.838], AUC_female_ = 0.606, 95% CI [0.435, 0.777]; both: AUC_male_ = 0.690, 95% CI [0.547, 0.837], AUC_female_ = 0.656, 95% CI [0.491, 0.821]; Supplementary Figure S6).

## 4 Discussion

This study investigated the abundance levels of CHIT1 and CHI3L1 in the TF of ALS and control patients, and compared their levels to those in serum, CSF, and additional clinical parameters.

As previously described, patients with ALS showed significantly reduced TF production compared to healthy controls, quantified by WL [16]. The finding of a strong positive correlation between WL and total protein concentration aligns with previous findings [24, 25]. This relationship highlights one of the methodological challenges of tear fluid biomarker studies. While normalization to total protein content is currently a widely used approach and facilitates comparison between samples with variable tear volumes, no consensus normalization strategy has been established, and validated reference proteins for tear fluid are lacking [15]. Furthermore, although reduced tear production may influence biomarker concentrations, it remains unclear to what extent the moderate reduction in WL observed in ALS alters tear protein composition, in contrast to the well‐described compositional differences between basal and reflex tears [26, 27]. Therefore, normalization to total protein content, which was used in this study, should be regarded as a pragmatic but imperfect approach.

We further examined the frequency of a 24 bp‐duplication polymorphism in the CHIT1 gene, which is present in about one third of the European population [23]. Although homozygotes were detected only among ALS patients in this cohort and the polymorphism showed a trend towards a higher frequency in this group, the difference did not reach statistical significance. Previous studies have reported varying findings regarding this association [13, 28]. The polymorphism is known to result in lower levels of CHIT1 in serum and CSF due to increased intracellular degradation [13, 14], which is confirmed in our data. Notably, we show that CHIT1 expression in TF is affected to the same extent by the respective genotype, suggesting that the impact of the polymorphism extends across different biofluids. Because of the largely even distribution of the polymorphism in our cohort, we chose neither to correct for the polymorphism nor to exclude homozygous carriers, an approach chosen by most other studies as well, although the genotype was only rarely analyzed.

Chitinases are established markers of neuroinflammation and are known to be increased in the CSF but not the serum of ALS patients [9, 29, 30], which was confirmed in this study. Importantly, this study, to the best of our knowledge, provides the first insight into the abundances of CHIT1 and CHI3L1 in TF. We show significantly elevated concentrations of CHI3L1 in the TF of ALS patients. CHIT1 levels exhibit the same trend, albeit missing significance. There was no significant correlation between CHIT1 and CHI3L1 abundances in TF and CSF.

Interestingly, CHIT1 and CHI3L1 levels were strongly correlated in both CSF and tear fluid, but not in serum. As both proteins are associated with neuroinflammatory responses and activated glial cells within the CNS, their concordant regulation in CSF is not unexpected and has been described before [29]. The preservation of this relationship in tear fluid may indicate that tear fluid reflects neuroinflammation‐associated processes more closely than blood. Importantly, the correlation between CSF and tear fluid CHI3L1 has been reported previously [31]. In contrast, the broader range of peripheral cellular sources of CHI3L1 compared to CHIT1 may contribute to the loss of correlation in serum [8, 32]. While the mechanisms linking CNS and tear fluid biomarker levels remain unclear, our findings further support tear fluid as a promising matrix for neuroinflammatory biomarker assessment.

Interestingly, CHIT1 and CHI3L1 in TF separately, as well as combined, showed better performance in distinguishing ALS patients from healthy controls using ROC‐curve analysis compared to serum. Here, CHI3L1 in TF alone showed the strongest diagnostic performance within TF and showed comparative performance to CSF. Previously, however, CHIT1 was shown to have better discriminating power than CHI3L1 in CSF [33]. One explanation for the observed inferiority of CHIT1 compared to CHI3L1 in TF in our study might be its low concentration in TF, which resulted in a third of the samples being below the limit of detection. Importantly, although the observed differences in CHI3L1 and CHIT1 levels between ALS and control subjects support the potential of tear fluid as a biomarker source, the substantial overlap between groups limits their discriminatory power at the individual patient level. Thus, these markers are currently better suited for investigating disease‐associated biological processes than for use as standalone diagnostic biomarkers.

Considering clinically relevant subgroups, we observed higher CHIT1 values in males in TF only, not in serum or CSF. In the male subgroup, CHIT1 values further correlated with the progression rate of ALS patients. This is in line with studies of CSF data where CHIT1 has been described as being associated with the progression rate [29, 30]. As no significant association between CHIT1 in TF and progression rate was observed in the overall ALS cohort, this finding should be considered exploratory and warrants validation in independent cohorts. Similarly, no significant correlations between CHIT1 or CHI3L1 levels in TF and disease duration or ALSFRS‐R score were observed. However, given the limited sample size, more subtle stage‐dependent associations cannot be excluded.

Additionally, both Chitinases in TF individually and in combination showed better classifier performance in males compared to females as well as to the whole cohort. Sex‐related differences in ALS have been frequently discussed, but the underlying biological mechanisms are still not completely understood [4, 34, 35]. Interestingly, males were shown to demonstrate stronger transcriptomic, miRNAomic, as well as proteomic deregulations in prefrontal cortex tissue, suggesting a more aggressive disease progression than in females [4]. Furthermore, biological differences in lacrimal gland physiology between males and females have been described [36], although whether these differences influence tear fluid CHIT1 or CHI3L1 concentrations is currently unknown. Alternatively, given the limited subgroup sizes, the observed sex‐specific associations may also reflect limited statistical power. Consequently, whether the differences observed in the present study reflect sex‐specific neuroinflammatory processes, characteristics of the tear fluid compartment, or statistical variation remains to be further studied.

Lastly, we investigated the additive discriminative power of serum NfL, a well‐described biomarker in ALS, in combination with chitinase values of the different biofluids. While the diagnostic accuracy of serum NfL did not increase using the serum abundances of the chitinases, values in CSF and, importantly, TF resulted in a modest increase in diagnostic performance. This finding may reflect the complementary biological information provided by NfL and chitinases, representing axonal damage and neuroinflammation, respectively. However, the diagnostic performance of serum NfL and the incremental value of the combined biomarker model should be interpreted with caution. The comparatively lower diagnostic accuracy of serum NfL in our cohort may, in part, reflect the heterogeneous control population, which included neurological disorders associated with elevated serum NfL concentrations. Nevertheless, these findings support the potential of combining serum NfL with TF chitinases as a minimally invasive approach that warrants further evaluation in larger prospective cohorts for longitudinal monitoring and disease stratification.

Our study has important limitations. First, this was a single‐center study with a relatively limited sample size. Although this reflects the rarity of ALS, the statistical power was limited, particularly for subgroup analyses. Due to the low concentration of CHIT1 in TF and the resulting limited detectability, a subset of patients' values could not be measured, and small differences among the patients were potentially missed. In addition, due to the retrospective cohort design, CSF samples collected during the same visit as serum and TF were only available for a limited group of patients. Normalization of tear fluid biomarkers remains an unresolved methodological challenge. Although total protein normalization, used in the current study, is common practice, it may not fully account for differences in tear secretion, underscoring the need for standardized normalization strategies. Furthermore, the heterogeneous composition of the control cohort may have influenced serum NfL concentrations and consequently the observed diagnostic performance of serum NfL and the combined biomarker model.

In conclusion, this study is, to the best of our knowledge, the first to investigate the abundance of chitinases, namely CHIT1 and CHI3L1, in the TF of ALS patients compared to healthy controls. This supports the potential of TF as a non‐invasively collectable biofluid, reflecting similar alterations as in CSF, which are crucially not detectable in blood. Further studies using more sensitive analytical techniques and larger, independent cohorts are needed to expand on these findings.

## Author Contributions

**Lara Wenz:** investigation, writing – original draft, visualization, methodology, writing – review and editing, formal analysis. **Lena‐Sophie Scholl:** methodology, writing – review and editing. **Nya Reinhardt:** investigation, writing – review and editing. **Ricarda von Heynitz:** investigation, writing – review and editing. **Vincent Gmeiner:** investigation, writing – review and editing. **Petra Rau:** investigation, writing – review and editing. **Philipp Jie Müller:** investigation, writing – review and editing. **Andreas W. Wolff:** formal analysis, writing – review and editing. **Emily Feneberg:** supervision, resources, writing – review and editing. **Antonia F. Demleitner:** conceptualization, investigation, formal analysis, methodology, supervision, writing – original draft, writing – review and editing. **Paul Lingor:** conceptualization, formal analysis, methodology, supervision, writing – review and editing, writing – original draft, resources.

## Funding

P.L. received funding from the DFG (German Research Foundation) under Germany's Excellence Strategy within the framework of the Munich Cluster for Systems Neurology (EXC 2145 SyNergy – ID 390857198). A.F.D. was supported by the Kommission für Klinische Forschung (KKF) of the TUM School for Medicine and Health (project H‐13).

## Ethics Statement

This study involved human participants and was approved by the Ethics review board of the Technical University of Munich (approval numbers: 9/15S, 2021‐473‐S‐KH). Participants gave informed consent to participate in the study before taking part.

## Conflicts of Interest

The authors declare no conflicts of interest.

## Supporting information


**Figure S1:** Correlation of the total protein concentration [μg/μl] in TF and wetting length [mm/5 min].
**Figure S2:** CHIT1 values in serum depending on the CHIT1 polymorphism.
**Figure S3:** Correlation of chitinase levels across all biofluids.
**Figure S4:** Correlation of chitinase values in TF with clinical parameters.
**Figure S5:** Correlation of NfL values in serum with chitinase levels in all biofluids.
**Figure S6:** ROC analysis of chitinase values in TF (ALS vs. controls), separated by sex.
**Table S1:** Characteristics of the CSF samples.
Supplementary Methods.

## Data Availability

The data that support the findings of this study are available from the corresponding authors upon reasonable request.

## References

[1] P. Masrori and P. Van Damme , “Amyotrophic Lateral Sclerosis: A Clinical Review,” European Journal of Neurology 27, no. 10 (2020): 1918–1929, 10.1111/ene.14393. PMC7540334 32526057

[2] D. R. Beers and S. H. Appel , “Immune Dysregulation in Amyotrophic Lateral Sclerosis: Mechanisms and Emerging Therapies,” Lancet Neurology 18, no. 2 (2019): 211–220, 10.1016/S1474-4422(18)30394-6. 30663610

[3] K. O'Neill , R. Shaw , I. Bolger , O. H. Tam , H. Phatnani , and M. Gale Hammell , “ALS Molecular Subtypes Are a Combination of Cellular and Pathological Features Learned by Deep Multiomics Classifiers,” Cell Reports 44, no. 3 (2025): 115402, 10.1016/j.celrep.2025.115402. PMC12011103 40067829

[4] L. Caldi Gomes , S. Hanzelmann , F. Hausmann , et al., “Multiomic ALS Signatures Highlight Subclusters and Sex Differences Suggesting the MAPK Pathway as Therapeutic Target,” Nature Communications 15, no. 1 (2024): 4893, 10.1038/s41467-024-49196-y. PMC11161513 38849340

[5] L. Vu , K. Garcia‐Mansfield , A. Pompeiano , et al., “Proteomics and Mathematical Modeling of Longitudinal CSF Differentiates Fast Versus Slow ALS Progression,” Annals of Clinical Translational Neurology 10, no. 11 (2023): 2025–2042, 10.1002/acn3.51890. PMC10647001 37646115

[6] L. Tzeplaeff , A. V. Jurs , C. Wohnrade , and A. F. Demleitner , “Unraveling the Heterogeneity of ALS‐A Call to Redefine Patient Stratification for Better Outcomes in Clinical Trials,” Cells 13, no. 5 (2024): 452, 10.3390/cells13050452. PMC10930688 38474416

[7] P. Shahim , G. Norato , N. Sinaii , et al., “Neurofilaments in Sporadic and Familial Amyotrophic Lateral Sclerosis: A Systematic Review and Meta‐Analysis,” Genes 15, no. 4 (2024): 496, 10.3390/genes15040496. PMC11050235 38674431

[8] N. Gaur , C. Perner , O. W. Witte , and J. Grosskreutz , “The Chitinases as Biomarkers for Amyotrophic Lateral Sclerosis: Signals From the CNS and Beyond,” Frontiers in Neurology 11 (2020): 377, 10.3389/fneur.2020.00377. PMC7267218 32536900

[9] A. Xu , Y. Luo , Y. Tang , et al., “Chitinases as a Potential Diagnostic and Prognostic Biomarker for Amyotrophic Lateral Sclerosis: A Systematic Review and Meta‐Analysis,” Neurological Sciences 45, no. 6 (2024): 2489–2503, 10.1007/s10072-024-07301-5. PMC11081993 38194198

[10] M. Dreger , R. Steinbach , M. Otto , M. R. Turner , and J. Grosskreutz , “Cerebrospinal Fluid Biomarkers of Disease Activity and Progression in Amyotrophic Lateral Sclerosis,” Journal of Neurology, Neurosurgery, and Psychiatry 93, no. 4 (2022): 422–435, 10.1136/jnnp-2021-327503. PMC8921583 35105727

[11] E. Bargagli , D. Bennett , C. Maggiorelli , et al., “Human Chitotriosidase: A Sensitive Biomarker of Sarcoidosis,” Journal of Clinical Immunology 33, no. 1 (2013): 264–270, 10.1007/s10875-012-9754-4. 22878841

[12] G. L. Chupp , C. G. Lee , N. Jarjour , et al., “Chitinase‐like Protein in the Lung and Circulation of Patients with Severe Asthma,” New England Journal of Medicine 357, no. 20 (2007): 2016–2027, 10.1056/NEJMoa073600. 18003958

[13] D. Gagliardi , M. Rizzuti , P. Masrori , et al., “Exploiting the Role of CSF NfL, CHIT1, and miR‐181b as Potential Diagnostic and Prognostic Biomarkers for ALS,” Journal of Neurology 271 (2024): 7557–7571, 10.1007/s00415-024-12699-1. PMC11588799 39340541

[14] A. Csongradi , I. T. Altorjay , G. A. Fulop , et al., “Chitotriosidase Gene Polymorphisms and Mutations Limit the Determination of Chitotriosidase Expression in Sarcoidosis,” Clinica Chimica Acta 513 (2021): 50–56, 10.1016/j.cca.2020.11.025. 33307063

[15] M. Gijs , N. van de Sande , C. Bonnet , et al., “A Comprehensive Scoping Review of Methodological Approaches and Clinical Applications of Tear Fluid Biomarkers,” Progress in Retinal and Eye Research 106 (2025): 101338, 10.1016/j.preteyeres.2025.101338. 39954936

[16] E. Luib , A. F. Demleitner , I. Cordts , et al., “Reduced Tear Fluid Production in Neurological Diseases: A Cohort Study in 708 Patients,” Journal of Neurology 271, no. 4 (2024): 1824–1836, 10.1007/s00415-023-12104-3. PMC10973005 38063868

[17] F. Maass , S. Rikker , V. Dambeck , et al., “Increased Alpha‐Synuclein Tear Fluid Levels in Patients With Parkinson's Disease,” Scientific Reports 10, no. 1 (2020): 8507, 10.1038/s41598-020-65503-1. PMC7244583 32444780

[18] A. F. Demleitner , L. C. Gomes , L. Wenz , et al., “An Exploratory Analysis of Differential Tear Fluid miRNAs in Patients With Parkinson's Disease and Atypical Parkinsonian Syndromes,” Molecular Neurobiology 62 (2025): 16397–16409, 10.1007/s12035-025-05252-2. PMC12559130 40758158

[19] M. Schmitz , S. Silva Correia , P. Hermann , et al., “Detection of Prion Protein Seeding Activity in Tear Fluids,” New England Journal of Medicine 388, no. 19 (2023): 1816–1817, 10.1056/NEJMc2214647. 37163630

[20] M. Gijs , N. Jorna , N. Datson , et al., “High Levels of Mutant Huntingtin Protein in Tear Fluid From Huntington's Disease Gene Expansion Carriers,” J Mov Disord 17, no. 2 (2024): 181–188, 10.14802/jmd.24014. PMC11082600 38379425

[21] L. S. Scholl , A. F. Demleitner , J. Riedel , et al., “Identification and Validation of a Tear Fluid‐Derived Protein Biomarker Signature in Patients With Amyotrophic Lateral Sclerosis,” Acta Neuropathologica Communications 13, no. 1 (2025): 187, 10.1186/s40478-025-02109-6. PMC12403336 40898360

[22] J. M. Shefner , A. Al‐Chalabi , M. R. Baker , et al., “A Proposal for New Diagnostic Criteria for ALS,” Clinical Neurophysiology 131, no. 8 (2020): 1975–1978, 10.1016/j.clinph.2020.04.005. 32387049

[23] R. G. Boot , G. H. Renkema , M. Verhoek , et al., “The Human Chitotriosidase Gene. Nature of Inherited Enzyme Deficiency,” Journal of Biological Chemistry 273, no. 40 (1998): 25680–25685, 10.1074/jbc.273.40.25680. 9748235

[24] M. Boerger , S. Funke , A. Leha , et al., “Proteomic Analysis of Tear Fluid Reveals Disease‐Specific Patterns in Patients With Parkinson's Disease‐A Pilot Study,” Parkinsonism & Related Disorders 63 (2019): 3–9, 10.1016/j.parkreldis.2019.03.001. 30876839

[25] M. Gijs , S. Arumugam , N. van de Sande , et al., “Pre‐Analytical Sample Handling Effects on Tear Fluid Protein Levels,” Scientific Reports 13, no. 1 (2023): 1317, 10.1038/s41598-023-28363-z. PMC9873914 36693949

[26] N. Perumal , S. Funke , D. Wolters , N. Pfeiffer , and F. H. Grus , “Characterization of Human Reflex Tear Proteome Reveals High Expression of Lacrimal Proline‐Rich Protein 4 (PRR4),” Proteomics 15, no. 19 (2015): 3370–3381, 10.1002/pmic.201400239. 26173177

[27] D. A. Dartt , “Neural Regulation of Lacrimal Gland Secretory Processes: Relevance in Dry Eye Diseases,” Progress in Retinal and Eye Research 28, no. 3 (2009): 155–177, 10.1016/j.preteyeres.2009.04.003156. PMC3652637 19376264

[28] P. Oeckl , “Differences in Inflammatory Profiles Between ALS and FTD,” Journal of Neurology, Neurosurgery, and Psychiatry 90, no. 1 (2019): 1, 10.1136/jnnp-2018-318868. 30224547

[29] A. G. Thompson , E. Gray , A. Bampton , D. Raciborska , K. Talbot , and M. R. Turner , “CSF Chitinase Proteins in Amyotrophic Lateral Sclerosis,” Journal of Neurology, Neurosurgery, and Psychiatry 90, no. 11 (2019): 1215–1220, 10.1136/jnnp-2019-320442. 31123140

[30] P. Steinacker , F. Verde , L. Fang , et al., “Chitotriosidase (CHIT1) is Increased in Microglia and Macrophages in Spinal Cord of Amyotrophic Lateral Sclerosis and Cerebrospinal Fluid Levels Correlate With Disease Severity and Progression,” Journal of Neurology, Neurosurgery, and Psychiatry 89, no. 3 (2017): 239–247, 10.1136/jnnp-2017-317138. 29142138

[31] J. Castillo‐Villalba , R. Gasque‐Rubio , L. Fores‐Toribio , et al., “Tear CHI3L1 in Multiple Sclerosis: A Non‐Invasive Biomarker Linked to Disease Progression,” Multiple Sclerosis and Related Disorders 112 (2026): 107319, 10.1016/j.msard.2026.107319. 42302565

[32] S. Ziatabar , J. Zepf , S. Rich , B. T. Danielson , P. I. Bollyky , and R. Stern , “Chitin, Chitinases, and Chitin Lectins: Emerging Roles in Human Pathophysiology,” Pathophysiology 25, no. 4 (2018): 253–262, 10.1016/j.pathophys.2018.02.005. 30266339

[33] L. Vu , J. An , T. Kovalik , T. Gendron , L. Petrucelli , and R. Bowser , “Cross‐Sectional and Longitudinal Measures of Chitinase Proteins in Amyotrophic Lateral Sclerosis and Expression of CHI3L1 in Activated Astrocytes,” Journal of Neurology, Neurosurgery, and Psychiatry 91, no. 4 (2020): 350–358, 10.1136/jnnp-2019-321916. PMC7147184 31937582

[34] J. A. Santiago , J. P. Quinn , and J. A. Potashkin , “Network Analysis Identifies Sex‐Specific Gene Expression Changes in Blood of Amyotrophic Lateral Sclerosis Patients,” International Journal of Molecular Sciences 22, no. 13 (2021): 7150, 10.3390/ijms22137150. PMC8269377 34281203

[35] B. J. Murdock , S. A. Goutman , J. Boss , S. Kim , and E. L. Feldman , “Amyotrophic Lateral Sclerosis Survival Associates With Neutrophils in a Sex‐Specific Manner,” Neurology(R) Neuroimmunology & Neuroinflammation 8, no. 2 (2021): e953, 10.1212/NXI.0000000000000953. PMC8057067 33531377

[36] S. Kastelan , K. Hat , Z. Tomic , T. Matejic , and N. Gotovac , “Sex Differences in the Lacrimal Gland: Implications for Dry Eye Disease,” International Journal of Molecular Sciences 26, no. 8 (2025): 3833, 10.3390/ijms26083833. PMC12028224 40332492

